# Estimation of Serum Vitamin B12 Levels Among the Elderly Population of Wardha City: A Cross-Sectional Study

**DOI:** 10.7759/cureus.28194

**Published:** 2022-08-20

**Authors:** Ranjana Sharma, Mayur B Wanjari, Arati Raut, Samruddhi Gujar, Ashna Gledina, Vaishnavi V Kantode, Pratiksha K Munjewar

**Affiliations:** 1 Medical Surgical Nursing, Smt. Radhikabai Meghe Memorial College of Nursing, Datta Meghe Institute of Medical Sciences, Wardha, IND; 2 Epidemiology and Public Health, Jawaharlal Nehru Medical College, Datta Meghe Institute of Medical Sciences (Deemed to be University), Wardha, IND

**Keywords:** biochemically, b12 deficiency, macroparticle immunosorbent assay, elderly, vitamin b12

## Abstract

Background

Globally, low vitamin B12 levels, undernutrition, and folic acid deficiency are more common among the geriatric population. Nevertheless, low vitamin serum levels may reveal a deficiency in the routine following of nutrition recommendations. In this study, we aimed to estimate the serum vitamin B12 levels among the elderly.

Methodology

This study was conducted in Wardha city among 90 elderly people over 60 years of age using a cross-sectional research design. Blood samples were utilized to estimate serum vitamin B12 levels by the macroparticle immunosorbent assay method using Abbott’s IMX system (Abbott Park, IL, USA). The data were analyzed using SPSS Statistics for Windows, version 24.0 (IBM Corp, Armonk, NY, USA). Numbers and percentages were used to represent qualitative characteristics. The mean (±SD) and median of a quantitative variable were used to compare groups using the analysis of variance test.

Results

In total, 57 (63.33%) of the elderly population had low (<110 pmol/L) vitamin B12 levels, 30 (33.33%) had medium (110-180 pmol/L) vitamin B12 levels, and three (3.34%) had high (>180 pmol/L) vitamin B12 levels. The mean was 168.11 ± 192.47 pmol/L for the estimation of the vitamin B12 level.

Conclusions

Untreated vitamin B12 deficiency is common among the elderly of both genders. However, there is no particular risk category for screening that can be recognized. Therefore, it is appropriate to screen the elderly biochemically. General practitioners play a key role in the initial vitamin B12 insufficiency diagnosis.

## Introduction

Vitamin B12 deficiency was considered a rare disease, but megaloblastic anemia makes it easier to diagnose. Vitamin B12 deficiency is common and often found in older people. Based on the diagnostic criteria, it affects an estimated 5-40% of older people. Small and carefully chosen samples have been utilized in numerous earlier studies to examine vitamin B12-related parameters in the elderly [1].

Due to symptoms and laboratory constraints, it is challenging to make a timely diagnosis of vitamin B12 insufficiency. Early warning indications can be undetectable or vague. To prevent irreversible damage, early detection is essential. Examining elderly patients with vague symptoms is a primary responsibility of general practitioners. People with known risk factors should be particularly concerned because typical signs and symptoms of vitamin B12 deficiency are frequently present [2].

Smoking, alcohol consumption, and vegetarianism are a few lifestyle choices that might contribute to vitamin B12 insufficiency. Metformin, gastrointestinal disorders, and medications lower gastric acid medications can make malabsorption worse. There is proof that various autoimmune illnesses are linked to pernicious anemia [3].

## Materials and methods

Study setting and design

This cross-sectional study was conducted in Wardha city located in Maharashtra (India) during July and August 2020. For this study, a local elderly population was selected.

Study population

In this study, the approach of purposive sampling was employed. In total, 90 individuals above the age of 60 years were chosen for this research. The elderly population that met the inclusion criteria, that was available at the time of the study, and was willing to participate was included in this study.

Data collection

The most important part of any investigation is gathering enough data to help address the main study topic, which is how much vitamin B12 the elderly consume. Blood was drawn to conduct a thorough examination. Each participant was handed a questionnaire on the first day and was asked to fill it out with details about their demographics, such as their age, gender, and health issues. The questionnaire was gathered shortly after being completed. Blood samples were utilized to confirm the estimate of serum vitamin B12 by the macroparticle immunosorbent assay method using Abbott’s IMX system (Abbott Park, IL, USA).

Sample collection and laboratory analysis

Venepuncture was used to collect 5 mL of blood from study participants, which was then injected further into the bare bulb. Blood samples were taken by a trained phlebotomist. The samples were delivered in airtight containers and sealed bags. The macroparticle immunosorbent assay test was performed after the samples arrived at the lab.

Statistical analysis

Utilizing the SPSS version 24 (IBM Corp., Armonk, NY, USA), the gathered data were examined. Demographic information was gathered using a questionnaire created specifically for the study by the research group. The questionnaire measured and rated the mean score. The association between the dependent (vitamin B12) and independent factors (socio-demographic profile) was examined using analysis of variance (ANOVA). The statistical significance level was attained at p-values of < 0.05 at a 95% confidence level (CI).

Ethical consideration

Each participant gave written informed permission after being fully told about the research and goals of the study. The participants received assurances of confidentiality and privacy. The Datta Meghe Institute of Medical Sciences (DMIMS(DU)/IEC/Dec-2020/8745) examined and accepted the study protocol.

## Results

Demographic characteristics of the study population

According to the demographic characteristics, the distribution of the elderly in terms of percentages is discussed here. Table 1 presents the sample attributes including age, gender, and health problem (Table 1).

**Table 1:** Demographic characteristics of the study population (n = 90).

Demographic variables	Number of subjects	Percentage (%)
Age (years)		
61–70	65	72.22
71–80	18	20.00
81–90	5	5.55
90 above	2	2.23
Gender		
Male	42	46.67
Female	48	53.33
Comorbidity		
Psychiatric disorder	2	2.22
Cardiovascular disorder	21	23.33
Cataract	48	53.33
Diabetes mellitus	05	5.56
Renal disorder	14	15.56

Estimation of the serum vitamin B12 among the elderly

The mean serum vitamin B12 among the elderly was 168.11 ± 192.47 pmol/L. Overall, 57 (63.33%) of the elderly population had low (<110 pmol/L), 30 (33.33%) had medium (110-180 pmol/L) vitamin B12 levels, and three (3.34%) had high (>180 pmol/L) vitamin B12 levels (Table 2).

**Table 2:** Serum vitamin B12 levels in the study population (n = 90).

Vitamin B12 levels	Number (%)	Mean (SD)	Standard error	95% CI
Low (<110)	57 (63.33%)	168.11 ± 192.47	20.29	127.79–208.42
Medium (110–180)	30 (33.33%)			
High (>180)	3 (3.34%)			

Association of the results with selected demographic variables

There were significant differences in the distribution of age in years and gender (p = 0.04; p = 0.03, respectively), and there was no difference between health problems (Table 3).

**Table 3:** Association of the study findings with selected demographic variables (n = 90).

Demographic variables	Number of the elderly	Vitamin B12 level (Mean ± SD)	F-value	P-value
Age (years)				
61–70	65	143.11 ± 148.02	2.89	p = 0.04 S, p < 0.05
71–80	18	243.00 ± 279.45		
81–90	5	115.20 ± 59.78		
90 above	2	439.00 ± 500.632		
Gender				
Male	42	204.48 ± 234.67	1.641	p = 0.03 S, p < 0.05
Female	48	136.29 ± 141.053		
Health problem				
Psychiatric disorder	2	99.00 ± 12.72	2.151	p = 0.08 NS, p < 0.05
Cardiac disorder	21	129.00 ± 126.77		
Cataract	48	220.00 ± 238.25		
Diabetes mellitus	05	84.40 ± 8.76		
Renal disorder	14	85.71 ± 17.89		

## Discussion

For individuals exhibiting equivocal total vitamin B12 concentrations (150-250 pmol/L), the measurement of tHcy, or holoTranscobalamin, has now been found to be an additional test [4]. Furthermore, hyperhomocysteinemia is common among the elderly. Renal efficiency, folate levels, vitamin B12 status, and plasma tHcy concentrations are primarily influenced by these factors. The efficacy of tHcy as a test performed for vitamin B12 insufficiency deteriorates with age because of the influence of reduced renal function and other illnesses2. We have used low holoTC in individuals exhibiting equivocal total vitamin B12 levels for diagnostic testing of vitamin B12 deficiency to get around the tHcy’s specificity issues [5].

Recent research has revealed that slight haptocorrin insufficiency is more widespread and may account for approximately 15% of the lower levels of vitamin B12 [6]. We have identified that the overdiagnosis of vitamin B12 insufficiency might have occurred due to our approach. Due to their normal tHcy and holoTC, five people in this sample may have received low levels of total vitamin B12. This is justifiable, in our opinion, because there are no hazardous side effects associated with vitamin B12 therapy, which makes overdiagnosis of vitamin B12 deficiency less damaging than underdiagnosis. Therefore, we believe that extra holoTC and tHcy measures are only helpful in people with marginal total vitamin B12 levels [7].

It appears that vitamin B12 insufficiency is significantly underdiagnosed. About 78% of the participants in our study cohort who had vitamin B12 insufficiency had never received a diagnosis. In clinical practice, greater vigilance is essential to avoid irreparable harm from delayed diagnosis. Consequently, screening the elderly who are asymptomatic has been recommended. Recognizing the early clinical signs or potential risk factors is an additional strategy. Meanwhile, few studies have examined the possible clinical associations besides cognitive impairment. They contend that the symptoms and signs of vitamin B12 insufficiency that have historically been connected to the illness are unrelated [8].

In this age group, deficiency of vitamin B12 was not correlated with smoking, drinking, or a vegetarian diet [6]. In younger individuals, these characteristics have been associated with a greater likelihood of vitamin B12 deficiency; however, in our experience, these associations have not been studied in older populations. The use of milk products was favorably linked with total vitamin B12 concentrations and was the norm rather than the exception among elderly Finns. The likelihood of lacking vitamin B12 quadrupled when milk products were avoided [9].

The findings of numerous earlier analyses of the relationships between cognitive performance and vitamin B12 levels change depending on the markers employed. We employed dementia diagnosis, the Mini-Mental State Examination (MMSE) score, and subjective cognitive deficits as relatively insensitive tests for mild cognitive dysfunction. As in numerous previous investigations, the MMSE score and total vitamin B12 or holoTC showed no association; however, tHcy showed a negative relationship. In addition, patients did not have a higher prevalence of vitamin B12 insufficiency with impaired subjective memory or low MMSE scores [10].

In addition, memory dysfunction indicators and a lower MMSE score have been linked to all of the vitamin B12 level measurements employed in recent research of individuals aged 75 years or older in the United Kingdom. Individuals with total vitamin B12 or holoTC concentrations in the lower quartile and tHcy or methylmalonic acid concentrations in the higher quartile were at higher risk for cognitive impairment [11]. Although depression was reported to be a cause and threat for vitamin B12 insufficiency in two earlier population-based investigations, the current analysis showed no connection between any indicators of vitamin B12 levels and depression [12].

Long-term studies are necessary to assess the usefulness of treating asymptomatic people. Aging raises the likelihood of vitamin B12 insufficiency, but there are no clear risk categories among the elderly [13]. Therefore, those aged 75 or older should, at the very least, undergo potential routine screening. We suggest measuring total vitamin B12 as a first-line investigation, with a confirmatory cutoff level of 150 pmol/L and an excluding cutoff limit of 250 pmol/L for vitamin B12 deficiency. It is advised that people with marginal total vitamin B12 concentrations (150-250 pmol/L) take further measurements of tHcy and holoTC. However, because the reference frame ranges for the tests vary, it is impossible to provide universal decision limits [14].

Limitations of the study

The limitations of the study include its small sample size and the reliability of the confirmation by Abbott’s IMX macroparticle immunosorbent assay.

## Conclusions

Vitamin B12 deficiency is most common in the general population and needs action regarding an early age detection of vitamin B12 that prevents severe causes. Regular health check-ups can avoid complications of vitamin B12. The rural and urban populations need to be aware of vitamin B12 deficiency. Health camps and awareness programs must be applied to improve awareness about this complication.
